# Supplementary material for: Reconciling emergences: An information-theoretic approach to identify causal emergence in multivariate data
Source: PLoS Comput Biol. 2020 Dec 21;16(12):e1008289. doi: 10.1371/journal.pcbi.1008289 (PMC7833221; doi:10.1371/journal.pcbi.1008289)
Supplement: S1 Appendix — (PDF) [file pcbi.1008289.s001.pdf]

S1 Appendix

1 Information decomposition in large multivariate systems

To formulate our theory of causal emergence for arbitrary order k, in Section *Partial information decomposition* we introduced definitions of kth-order synergy and unique information. In this appendix we complement these with a matching definition of kth-order redundancy, and show that these provide a full-fledged information decomposition for any $k = \{1, \dots, n-1\}$. For completeness, we present all definitions and examples here – including those that were necessary for the exposition of the main text and were previously presented in Section *Partial information decomposition*.

We begin by (re-)introducing the notion of kth-order synergy between n variables, defined as

$$\texttt{Syn}^{(k)}(\boldsymbol{X};Y) \coloneqq \sum_{\boldsymbol{\alpha}\in\mathcal{S}^{(k)}} I_\partial^{\boldsymbol{\alpha}}(\boldsymbol{X};Y) \ ,$$

with $\mathcal{S}^{(k)} = \{\{\alpha_1, \dots, \alpha_L\} \in \mathcal{A} : |\alpha_j| > k, \forall j = 1, \dots, L\}$. Intuitively, $\texttt{Syn}^{(k)}(\boldsymbol{X};Y)$ corresponds to the information about the target that is provided by the whole $\boldsymbol{X}$ but is not contained in any set of k or less parts when considered separately from the rest. Accordingly, $\mathcal{S}^{(k)}$ only contains collections of more than k sources. For example, for $n = 2$ we obtain the standard synergy $\mathcal{S}^{(1)} = \{\{12\}\}$, and for $n = 3$ we have $\mathcal{S}^{(1)} = \{\{12\}, \{13\}, \{23\}, \{12\}\{13\}, \{12\}\{23\}, \{13\}\{23\}, \{12\}\{13\}\{23\}, \{123\}\}$.

Similarly, the kth-order unique information of $\boldsymbol{X}^\beta$ with $\beta \subset [n]$ is calculated as

$$\texttt{Un}^{(k)}(\boldsymbol{X}^\beta;Y|\boldsymbol{X}^{-\beta}) \coloneqq \sum_{\boldsymbol{\alpha}\in\mathcal{U}^{(k)}(\beta)} I_\partial^{\boldsymbol{\alpha}}(\boldsymbol{X};Y) \ ,$$

with $\mathcal{U}^{(k)}(\beta) = \{\boldsymbol{\alpha} \in \mathcal{A} : \beta \in \boldsymbol{\alpha}, \forall \alpha \in \boldsymbol{\alpha} \setminus \beta, \alpha \subseteq [n] \setminus \beta, |\alpha| > k\}$, and $\boldsymbol{X}^{-\beta}$ being all the variables in $\boldsymbol{X}$ the indices of which are not in β. This corresponds to all the atoms where β is the only source of size k or less – which, importantly, is in general not just I_∂^β. Intuitively, this is the information that $\boldsymbol{X}^\beta$ has access to and no other subset of parts has access to *on its own* (although bigger groups of other parts may). And again, for $n = 2$ we recover $\mathcal{U}^{(1)}(\{i\}) = \{\{i\}\}$, and for $n = 3$ we have e.g. $\mathcal{U}^{(1)}(\{1\}) = \{\{1\}, \{1\}\{23\}\}$.

Finally, the kth-order redundancy is given by

$$\texttt{Red}^{(k)}(\boldsymbol{X};Y) \coloneqq \sum_{\boldsymbol{\alpha}\in\mathcal{R}^{(k)}} I_\partial^{\boldsymbol{\alpha}}(\boldsymbol{X};Y) \ ,$$

with $\mathcal{R}^{(k)} = \{\boldsymbol{\alpha} \in \mathcal{A} : \exists i \neq j, |\alpha_i|, |\alpha_j| \leq k\}$. Intuitively, $\texttt{Red}^{(k)}(\boldsymbol{X};Y)$ is the information that is held by at least two different groups of size k or less. Again, in the $n = 2$ case we recover the standard redundancy $\mathcal{R}^{(1)} = \{\{1\}\{2\}\}$; and as an example for $n = 3$ we have $\mathcal{R}^{(1)} = \{\{1\}\{2\}, \{1\}\{3\}, \{2\}\{3\}, \{1\}\{2\}\{3\}\}$.

With the definitions above, we can build a coarse-grained PID which generalises the well-known construction for $n = 2$. This allows us to formulate decompositions with a small number of atoms that scale gracefully with system size, and, more interestingly, preserve the intuitive meaning that synergy, redundancy, and unique information have for $n = 2$.

Lemma 1. *The kth-order synergy, redundancy, and unique information defined above provide an exact decomposition of mutual information:*

$$I(\boldsymbol{X};Y) = \texttt{Red}^{(k)}(\boldsymbol{X};Y) + \texttt{Syn}^{(k)}(\boldsymbol{X};Y) + \sum_{\substack{\beta \subset [n]: \\ |\beta| \leq k}} \texttt{Un}^{(k)}(\boldsymbol{X}^{\beta};Y|\boldsymbol{X}^{-\beta}) \ . \qquad (1)$$

Proof. We will prove this by showing that the sets $\mathcal{R}^{(k)}$, $\mathcal{S}^{(k)}$ and $\mathcal{U}^{(k)}(\beta)$ are a partition of $\mathcal{A}$. We will do this in two steps: first, we show that their intersection is empty; and second, that their union is $\mathcal{A}$.

Let us show that the intersections between every pair of sets is empty:

- $\mathcal{R}^{(k)} \bigcap \mathcal{S}^{(k)} = \varnothing$, since if $\boldsymbol{\alpha} \in \mathcal{R}^{(k)}$ it must contain at least one $\alpha \in \boldsymbol{\alpha} : |\alpha| \leq k$, and therefore $\boldsymbol{\alpha} \notin \mathcal{S}^{(k)}$.
- $\mathcal{U}^{(k)}(\gamma) \bigcap \mathcal{U}^{(k)}(\beta) = \varnothing$ if and only if $\gamma \neq \beta$, since every $\boldsymbol{\alpha} \in \mathcal{U}^{(k)}(\gamma)$ has either no other elements apart from γ (in which case $\beta \notin \boldsymbol{\alpha}$ and thus $\boldsymbol{\alpha} \notin \mathcal{U}^{(k)}(\beta)$), or other elements of cardinality greater than k (in which case, again, $\beta \notin \boldsymbol{\alpha}$ and thus $\boldsymbol{\alpha} \notin \mathcal{U}^{(k)}(\beta)$).
- $\mathcal{S}^{(k)} \bigcap \mathcal{U}^{(k)}(\beta) = \varnothing$ for all $|\beta| \leq k$, since every $\boldsymbol{\alpha} \in \mathcal{U}^{(k)}(\beta)$ contains at least one element with cardinality less than or equal to k (specifically, β), and therefore $\boldsymbol{\alpha} \notin \mathcal{S}^{(k)}$.
- $\mathcal{R}^{(k)} \bigcap \mathcal{U}^{(k)}(\beta) = \varnothing$ for all $|\alpha| \leq k$, since every $\boldsymbol{\alpha} \in \mathcal{R}^{(k)}$ contains at least two sets with cardinality less than or equal to k, while by the definition of $\mathcal{U}^{(k)}(\beta)$ every element other than β must have cardinality greater than k, and thus $\boldsymbol{\alpha} \notin \mathcal{U}^{(k)}(\beta)$.

This concludes the first part of the proof. Next, we need to prove that the union of those sets is indeed $\mathcal{A}$. We will show this by proving that every $\boldsymbol{\alpha} \in \mathcal{A}$ is in one of those sets:

- If $\nexists \alpha \in \boldsymbol{\alpha} : |\alpha| \leq k$, then $\boldsymbol{\alpha} \in \mathcal{S}^{(k)}$.
- If there is exactly one $\alpha \in \boldsymbol{\alpha} : |\alpha| \leq k$, then $\boldsymbol{\alpha} \in \mathcal{U}^{(k)}(\alpha)$.
- If there are more than one $\alpha \in \boldsymbol{\alpha} : |\alpha| \leq k$, then $\boldsymbol{\alpha} \in \mathcal{R}^{(k)}$.

□

The two possible decompositions for $n = 3$, together with the standard PID lattice, are shown in Figure A.

2 Properties of high-order PI atoms

As discussed in Section *A formal theory of causal emergence*, our theory does not depend on a specific functional form of PID. Instead, it applies to any PID that satisfies the following properties:

- *Deterministic equality*: if there exists a function $f(\cdot)$ such that $X^i = f(X^j)$ with $i \neq j$, then the information decomposition of $\boldsymbol{X}$ is isomorphic to that of $\boldsymbol{X}^{-j}$ (see below).
- *Non-negativity*: $\texttt{Syn}^{(k)}(\boldsymbol{X};Y) \geq 0$, and $\min\{I(\boldsymbol{Z};Y), I(\boldsymbol{Z};Y|\boldsymbol{X})\} \geq \texttt{Un}^{(k)}(\boldsymbol{Z};Y|\boldsymbol{X}) \geq 0$.

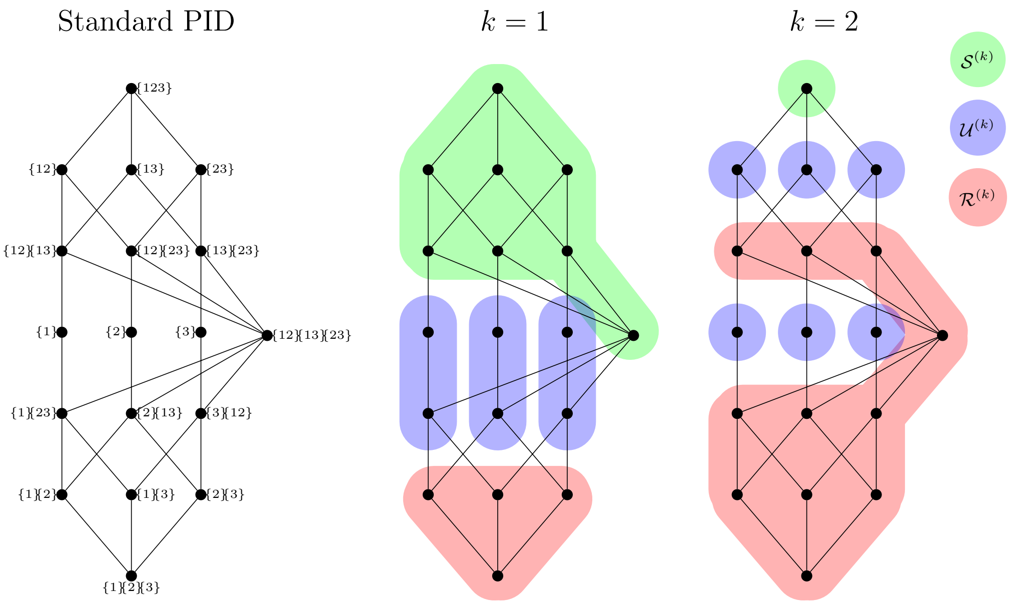

Fig A. Coarse-grained partial information decomposition of order k. (*left*) Standard PID lattice for $n = 3$, shown for reference. Node labels are omitted from the other lattices for clarity. *(middle)* Coarse-graining for $k = 1$, superimposed on the PID lattice. (*right*) Coarse-graining for $k = 2$. For both values of k, Lemma 1 guarantees that the kth-order atoms provide an exact decomposition of mutual information.

- *Source data processing inequality*: $\texttt{Un}^{(k)}(\boldsymbol{W}; Y|\boldsymbol{X}) \leq \texttt{Un}^{(k)}(\boldsymbol{Z}; Y|\boldsymbol{X})$ for all $\boldsymbol{W} - \boldsymbol{Z} - (\boldsymbol{X}, Y)$ Markov chains.

To formulate the causal decoupling and downward causation indices in Section *A taxonomy of emergence*, we make use of ΦID, a recent extension of PID to multi-target settings [1]. As with PID, our theory does not require a particular functional form of ΦID, only the following property:

- $\sum_{|\alpha|=k} \texttt{Syn}^{(k)}(\boldsymbol{X}_t; \boldsymbol{X}^{\alpha}_{t'}) \geq \mathcal{D}^{(k)}(\boldsymbol{X}_t; \boldsymbol{X}_{t'}) \geq \texttt{Syn}^{(k)}(\boldsymbol{X}_t; \boldsymbol{X}^{\beta}_{t'})$ for all $|\beta| = k$.

Finally, these properties are required to formulate the practical criteria for emergence in Section *A taxonomy of emergence*:

- *Whole-minus-sum*: $\texttt{Syn}^{(k)}(\boldsymbol{X}; Y) \geq I(\boldsymbol{X}; Y) - \sum_{|\alpha|=k} I(\boldsymbol{X}^{\alpha}; Y)$.
- *Target data processing inequality*: for all $\boldsymbol{X} - Y - U$ Markov chains, $\texttt{Syn}^{(k)}(\boldsymbol{X}; U) \leq \texttt{Syn}^{(k)}(\boldsymbol{X}; Y)$.

For completeness, we present a precise definition of the deterministic equality property, as previously introduced in the PID literature [2].

Definition 1. *A PID satisfies* deterministic equality *if* $I^{\boldsymbol{\alpha}}_{\partial}(\boldsymbol{X}; Y) = I^{g_j(\boldsymbol{\alpha})}_{\partial}(\boldsymbol{X}^{-j}; Y)$ *for all* $\boldsymbol{\alpha} \in \mathcal{A}$ *whenever there exists a function* $f(\cdot)$ *such that* $f(X^j) = X^i$ *with* $i \neq j$*, with* $g_j(\boldsymbol{\alpha})$ *removes* j *from all the sets of indices in* $\boldsymbol{\alpha}$*.*

It is direct to check that a number of well-known information decompositions, including the Minimum Mutual Information PID [3], and the corresponding ΦID [1], satisfy these requirements.

With these properties at hand we can prove the following results, used in Sections *Defining causal emergence* and *A taxonomy of emergence*:

Lemma 2. *If $X^{n+1} = \boldsymbol{X}$, then the following holds:*

$$\texttt{Syn}^{(k)}(\boldsymbol{X}; Y) = \texttt{Un}^{(k)}(X^{n+1}; Y|\boldsymbol{X}) \ . \tag{2}$$

Above, the second term corresponds to a PID over a system of $n+1$ elements.

Proof. Begin by considering the PID of n sources $X^1, ..., X^n$ on the lattice $\mathcal{A}^n$, and define its set $\mathcal{S}^{(k)}$ as above. Now we add an additional $n+1^{\text{st}}$ variable that is simply all of them concatenated, $X_{n+1} = \boldsymbol{X}^n$, and build a PID on the lattice $\mathcal{A}^{n+1}$. In the following, we consider the set $\mathcal{S}^{(k)}$ to belong to the lattice $\mathcal{A}^n$, and the set $\mathcal{U}^{(k)}(\beta)$ to belong to the lattice $\mathcal{A}^{n+1}$. To prove the lemma we need four ingredients, which we provide in the four paragraphs below.

1. First, note that the nodes in $\mathcal{A}^{n+1}$ that precede $\{n+1\}$ are those in $\mathcal{A}^n$, but with the singleton $\{n+1\}$ appended to them. More specifically, the mapping $f : \mathcal{A}^n \to \mathcal{A}^{n+1}$ of the form $f(\boldsymbol{\alpha}) = \boldsymbol{\alpha} \cup \{\{n+1\}\}$ is such that for any $\boldsymbol{\alpha} \in \mathcal{A}^n$ then $f(\boldsymbol{\alpha}) \prec \{\{n+1\}\}$. Additionally, due to the properties of the partial order, $\boldsymbol{\alpha} \preceq \boldsymbol{\alpha}'$ if and only if $f(\boldsymbol{\alpha}) \preceq f(\boldsymbol{\alpha}')$. This shows that $\mathcal{A}^n$ is isomorphic to a sublattice of $\mathcal{A}^{n+1}$.

2. Next, by the *deterministic equality* property it is direct to check that $I_\cap^{f(\boldsymbol{\alpha})} = I_\cap^{\boldsymbol{\alpha}}$. Since this equality holds for all $\boldsymbol{\alpha} \in \mathcal{A}^n$, then applying a Möbius inversion we directly obtain that $I_\partial^{f(\boldsymbol{\alpha})} = I_\partial^{\boldsymbol{\alpha}}$.

3. Additionally, by construction of $\mathcal{U}^{(k)}$ and $\mathcal{S}^{(k)}$, for all $\boldsymbol{\gamma} \in \mathcal{S}^{(k)}$ one has $f(\boldsymbol{\gamma}) \in \mathcal{U}^{(k)}(\{n+1\})$. In other words, the set $\mathcal{U}^{(k)}(\{n+1\})$ includes all atoms in $\mathcal{S}^{(k)}$, plus $\{n+1\}$.

4. Finally, note that the node $\{12...n\}\{n+1\}$ is the only direct predecessor of $\{n+1\}$, since there exists no node $\boldsymbol{\beta} \in \mathcal{A}^{n+1}$ such that $\boldsymbol{\beta} \prec \{n+1\}$ and not $\boldsymbol{\beta} \preceq \{12...n\}\{n+1\}$. By the *deterministic equality* property $I_\cap^{\{12...n\}\{n+1\}} = I_\cap^{\{12...n\}}$ and, therefore, $I_\partial^{\{n+1\}} = 0$.

With all of these, it is direct to see that

$$\begin{aligned} \texttt{Un}^{(k)}(\boldsymbol{X}^n; Y|\boldsymbol{X}) &= \sum_{\boldsymbol{\alpha} \in \mathcal{U}^{(k)}(\{n+1\})} I_\partial^{\boldsymbol{\alpha}}(\boldsymbol{X}; Y) \\ &= \sum_{\boldsymbol{\alpha} \in \mathcal{S}^{(k)}} I_\partial^{\boldsymbol{\alpha}}(\boldsymbol{X}; Y) = \texttt{Syn}^{(k)}(\boldsymbol{X}; Y) \ . \end{aligned}$$

□

Corollary 1. *If $X^{n+1} = \boldsymbol{X}$ and $Y^{n+1} = \boldsymbol{Y}$, then the following holds:*

$$\mathcal{G}^{(k)}(\boldsymbol{X}; \boldsymbol{Y}) = \texttt{Un}^{(k)}(X^{n+1}; Y^{n+1}|\boldsymbol{X}, \boldsymbol{Y}) \tag{3}$$

Above, the second term corresponds to a ΦID over a system of $n+1$ elements.

Proof. Follows from a direct ΦID extension to the proof of Lemma 2, by formulating a decomposition

$$I(\boldsymbol{X}; \boldsymbol{Y}) = \sum_{\boldsymbol{\alpha}, \boldsymbol{\beta} \in \mathcal{A}^n} I_\partial^{\boldsymbol{\alpha} \to \boldsymbol{\beta}}(\boldsymbol{X}; \boldsymbol{Y}) \ ,$$

and applying the proof above to both $\boldsymbol{\alpha}$ and $\boldsymbol{\beta}$. Strictly speaking, this also requires a natural multi-target extension of the Deterministic Equality property, namely that $I_\cap^{\boldsymbol{\alpha} \to \boldsymbol{\beta}}(\boldsymbol{X}; \boldsymbol{Y}) = I_\cap^{\boldsymbol{\alpha} \to \boldsymbol{\beta}}(\boldsymbol{X}^{-j}; \boldsymbol{Y})$ if $X^i = f(X^j)$ with $j \neq i$, for any $\boldsymbol{\beta} \in \mathcal{A}^n$; as well as the symmetric $I_\cap^{\boldsymbol{\alpha} \to \boldsymbol{\beta}}(\boldsymbol{X}; \boldsymbol{Y}) = I_\cap^{\boldsymbol{\alpha} \to \boldsymbol{\beta}}(\boldsymbol{X}; \boldsymbol{Y}^{-j})$ if $Y^i = f(Y^j)$ with $j \neq i$, for any $\boldsymbol{\alpha} \in \mathcal{A}^n$. □

3 Mathematical properties of causal emergence

Proof of Lemma 1. The first property can be easily proven by noting that there can be no synergy in a univariate system: i.e. if $n = 1$, then $\mathcal{S}^{(k)} = \varnothing$ and therefore $\mathtt{Syn}^{(k)}(\boldsymbol{X}_t; \boldsymbol{X}_{t'}) = 0$.

To prove the second property, let us assume that there exists a function $g(\cdot)$ such that $g(X_t^j) = V_t$ for some j. Then, one can show that

$$\mathtt{Un}^{(1)}(V_t; \boldsymbol{X}_{t'} | \boldsymbol{X}_t) \leq I(V_t; \boldsymbol{X}_{t'} | \boldsymbol{X}_t) \tag{4}$$

$$\leq I(V_t; \boldsymbol{X}_{t'} | X_t^j) \tag{5}$$

$$\leq H(V_t | X_t^j) \tag{6}$$

$$= 0 \ , \tag{7}$$

where (4) is due to the non-negativity property of $\mathtt{Un}^{(k)}$ introduced in Section *Partial information decomposition*. This shows that V_t cannot exhibit emergent behaviour.

□

Proof of Theorem 1. If $\mathtt{Syn}^{(k)}(\boldsymbol{X}_t; \boldsymbol{X}_{t'}) > 0$, then by Lemma 2 it is clear that the feature $V_t = \boldsymbol{X}_t$ exhibits causal emergence.

To prove the converse, note that all supervenient features follow the Markov chain structure $V_t - \boldsymbol{X}_t - \boldsymbol{X}_{t'}$. Therefore, for any supervenient feature $V_t = f(\boldsymbol{X}_t)$ the following holds:

$$0 \leq \mathtt{Un}^{(k)}(V_t; \boldsymbol{X}_{t'} | \boldsymbol{X}_t) \leq \mathtt{Un}^{(k)}(\boldsymbol{X}_t; \boldsymbol{X}_{t'} | \boldsymbol{X}_t) \tag{8}$$

$$= \mathtt{Syn}(\boldsymbol{X}_t; \boldsymbol{X}_{t'}) \ , \tag{9}$$

where (8) is due to the data processing inequality of the unique information (c.f. Section *Partial information decomposition*), and (9) is due to Lemma 2. Using this result, is clear that if $\mathtt{Syn}^{(k)}(\boldsymbol{X}_t; \boldsymbol{X}_{t'}) = 0$ then $\mathtt{Un}^{(k)}(V_t; \boldsymbol{X}_{t'} | \boldsymbol{X}_t) = 0$ for any superventient feature V_t. □

The above proof implies that the system has emergent behaviour if and only if the system as a whole seen as a feature (i.e. $V_t = \boldsymbol{X}_t$) is causally emergent. Note that the fact that this trivial feature is helpful for detecting the presence of emergence doesn't imply that it is an appropriate way of representing it, as in most cases also carries non-interesting information, and in practice one may be interested in features that exhibit emergence but are shorter to describe than the microstate of the system, i.e. $H(V_t) < H(\boldsymbol{X}_t)$.

Proof of Theorem 2. Let us first assume that there exists a supervenient feature V_t such that $\mathtt{Un}^{(k)}(V_t; \boldsymbol{X}_{t'}^{\alpha} | \boldsymbol{X}) > 0$ for some $\alpha : |\alpha| = k$. Then, one can find that

$$0 < \mathtt{Un}^{(k)}(V_t; \boldsymbol{X}_{t'}^{\alpha} | \boldsymbol{X}_t)$$

$$\leq \mathtt{Un}^{(k)}(\boldsymbol{X}_t; \boldsymbol{X}_{t'}^{\alpha} | \boldsymbol{X}_t) \tag{10}$$

$$= \mathtt{Syn}^{(k)}(\boldsymbol{X}_t; \boldsymbol{X}_{t'}^{\alpha}) \tag{11}$$

$$\leq \mathcal{D}^{(k)}(\boldsymbol{X}_t; \boldsymbol{X}_{t'}) \ . \tag{12}$$

Above, (10) is a consequence of the data processing inequality of the unique information, (11) comes from Lemma 2, and (12) is from the properties of $\mathcal{D}^{(k)}$ stated in Section *A taxonomy of emergence.*

To prove the converse, let us assume that all supervenient features V_t satisfy $\mathtt{Un}^{(k)}(V_t; \boldsymbol{X}_{t'}^\alpha | \boldsymbol{X}_t) = 0$ for all k and $|\alpha| = k$. In particular, this is true for the feature $V_t = \boldsymbol{X}_t$. Then, another application of Lemma 2 shows that

$$\begin{aligned} \mathcal{D}^{(k)}(\boldsymbol{X}_t; \boldsymbol{X}_{t'}) &\leq \sum_{|\alpha|=k} \mathtt{Syn}^{(k)}(\boldsymbol{X}_t; \boldsymbol{X}_{t'}^\alpha) \qquad (13) \\ &= \sum_{|\alpha|=k} \mathtt{Un}^{(k)}(\boldsymbol{X}_t; \boldsymbol{X}_{t'}^\alpha | \boldsymbol{X}_t) \\ &= 0 \ . \end{aligned}$$

Above, (13) is a consequence of the properties of $\mathcal{D}^{(k)}$.

□

Proof of Theorem 3. We begin by proving that a system has a causally decoupled feature iff $\mathcal{G}^{(k)}(\boldsymbol{X}_t; \boldsymbol{X}_{t'}) > 0$. This proof follows a similar structure to that of Theorem 2 for downward causation.

Let us first assume that there exists a supervenient feature V_t with $\mathtt{Un}^{(k)}(V_t; V_{t'} | \boldsymbol{X}_t, \boldsymbol{X}_{t'}) > 0$. Then,

$$\begin{aligned} 0 &< \mathtt{Un}^{(k)}(V_t; V_{t'} | \boldsymbol{X}_t, \boldsymbol{X}_{t'}) \\ &\leq \mathtt{Un}^{(k)}(\boldsymbol{X}_t; \boldsymbol{X}_{t'} | \boldsymbol{X}_t, \boldsymbol{X}_{t'}) \qquad (14) \\ &= \mathcal{G}^{(k)}(\boldsymbol{X}_t; \boldsymbol{X}_{t'}) \ . \qquad (15) \end{aligned}$$

Above, (14) can be obtained through a combination of the data processing inequalities of the unique information and the synergy, as well as Lemma 2; and (15) is an application of Corollary 1.

To prove the converse, assume that for all supervenient features $\mathtt{Un}^{(k)}(V_t; V_{t'} | \boldsymbol{X}_t, \boldsymbol{X}_{t'}) = 0$. As before, this also includes $V_t = \boldsymbol{X}_t$. Therefore, applying Corollary 1 we arrive at $\mathcal{G}^{(k)}(\boldsymbol{X}_t; \boldsymbol{X}_{t'}) = \mathtt{Un}^{(k)}(\boldsymbol{X}_t; \boldsymbol{X}_{t'} | \boldsymbol{X}_t, \boldsymbol{X}_{t'}) = 0$, which concludes the proof.

We now move on to prove the results on perfectly causally decoupled systems, where $\mathcal{G}^{(k)}(\boldsymbol{X}_t; \boldsymbol{X}_{t'}) > 0$ and $\mathcal{D}^{(k)}(\boldsymbol{X}_t; \boldsymbol{X}_{t'}) = 0$. As $\mathtt{Syn}^{(k)}(\boldsymbol{X}_t; \boldsymbol{X}_{t'}) = \mathcal{G}^{(k)}(\boldsymbol{X}_t; \boldsymbol{X}_{t'}) + \mathcal{D}^{(k)}(\boldsymbol{X}_t; \boldsymbol{X}_{t'}) > 0$, thanks to Theorem 1 this is equivalent to the existence of at least one emergent feature V_t. Additionally, due to Theorem 2, $\mathcal{D}^{(k)}(\boldsymbol{X}_t; \boldsymbol{X}_{t'}) = 0$ is equivalent to $\mathtt{Un}^{(k)}(V_t; \boldsymbol{X}_{t'}^\alpha | \boldsymbol{X}_t) = 0$ for all emergent features and $\alpha : |\alpha| = k$

□

4 Mathematical properties of emergence criteria

In this appendix we provide the necessary proofs linking the practical criteria for emergence in Eqs. (10a-10c) from the main text with the definitions in Section *A formal theory of causal emergence*. For completeness, we provide formulae of Ψ, Δ, and Γ for arbitrary emergence order k:

$$\Psi_{t,t'}^{(k)}(V) \coloneqq I(V_t; V_{t'}) - \sum_{|\alpha|=k} I(\boldsymbol{X}_t^\alpha; V_{t'}) \ , \qquad (16a)$$

$$\Delta_{t,t'}^{(k)}(V) \coloneqq \max_{|\alpha|=k} \left(I(V_t; \boldsymbol{X}_{t'}^\alpha) - \sum_{|\beta|=k} I(\boldsymbol{X}_t^\beta; \boldsymbol{X}_{t'}^\alpha) \right) \ , \qquad (16b)$$

$$\Gamma_{t,t'}^{(k)}(V) \coloneqq \max_{|\alpha|=k} I(V_t; \boldsymbol{X}_{t'}^\alpha) \ . \qquad (16c)$$

Proof of Proposition 1. To start, note that a direct calculation shows that

$$\Psi^{(k)}_{t,t'}(V) \leq I(\boldsymbol{X}_t; V_{t'}) - \sum_{|\alpha|=k} I(\boldsymbol{X}^\alpha_t; V_{t'}) \tag{17}$$

$$\leq \texttt{Syn}^{(k)}(\boldsymbol{X}_t; V_{t'}) \tag{18}$$

$$\leq \texttt{Syn}^{(k)}(\boldsymbol{X}_t; \boldsymbol{X}_{t'}) \ . \tag{19}$$

Above, (17) is due to the data processing inequality applied over the Markov chain $V_t - \boldsymbol{X}_t - \boldsymbol{X}_{t'} - V_{t'}$; (18) is due to the whole-minus-sum property of the synergy; and (19) is due to the data processing inequality of the synergy. Therefore, it is clear that if $\Psi^{(k)}_{t,t'}(V) > 0$ for some feature V_t then $\texttt{Syn}^{(k)}(\boldsymbol{X}_t; \boldsymbol{X}_{t'}) > 0$. This, combined with Theorem 1, guarantees that the system exhibits causal emergence.

To check the condition for downward causation, a direct calculation shows that, for some $|\alpha| = k$,

$$\Delta^{(k)}_{t,t'}(V) \leq I(\boldsymbol{X}_t; \boldsymbol{X}^\alpha_{t'}) - \sum_{|\boldsymbol{\beta}|=k} I(\boldsymbol{X}^\beta_t; \boldsymbol{X}^\alpha_{t'}) \tag{20}$$

$$\leq \texttt{Syn}^{(k)}(\boldsymbol{X}_t; \boldsymbol{X}^\alpha_{t'}) \tag{21}$$

$$\leq \mathcal{D}^{(k)}(\boldsymbol{X}_t; \boldsymbol{X}_{t'}) \ . \tag{22}$$

Above, (20) is due to the data processing inequality applied over the Markov chain $V_t - \boldsymbol{X}_t - \boldsymbol{X}^\alpha_{t'}$; (21) to the whole-minus-sum property of the synergy; and (22) to the properties of $\mathcal{D}^{(k)}$. From here, is clear that if $\Delta^{(k)}_{t,t'}(V) > 0$ for some feature V_t then $\mathcal{D}^{(k)}(\boldsymbol{X}_t; \boldsymbol{X}_{t'}) > 0$. This, combined with Theorem 1, guarantees that the system exhibits downward causation.

Finally, for the condition for causal decoupling it is sufficient to note that

$$\Gamma^{(k)}_{t,t'}(V) = \max_{|\alpha|=k} I(V_t; \boldsymbol{X}^\alpha_{t'}) \tag{23}$$

$$\geq \texttt{Un}^{(k)}(V_t; \boldsymbol{X}^\alpha_{t'} | \boldsymbol{X}_t) \geq 0 \ , \tag{24}$$

where the inequality is due to the bounds of the unique information. □

Proof of Proposition 2. Let us consider V_t a k-synergistic observable. Due to stationarity, the fact that $V_t \in \mathcal{C}_k(\boldsymbol{X}_t)$ implies that $V_{t'} \in \mathcal{C}_k(\boldsymbol{X}_{t'})$. Using this fact, and noting that $V_t - \boldsymbol{X}_t - \boldsymbol{X}_{t'} - V_{t'}$ is a Markov chain, it is direct to check that

$$\mathcal{G}^{(k)}_\star(\boldsymbol{X}_t; \boldsymbol{X}_{t'}) \geq I(V_t; V_{t'}) > 0 \ , \tag{25}$$

Additionally, since $\mathcal{D}^{(k)}_\star(\boldsymbol{X}_t; \boldsymbol{X}_{t'}) \geq 0$, Eq. (25) implies that $\texttt{Syn}^{(k)}_\star(\boldsymbol{X}_t; \boldsymbol{X}_{t'}) > 0$, proving the desired result. □

5 Simulation details

Let us focus first on the Game of Life (GoL) simulations illustrated by Figure 4 in the main text. For the initial state, two particles of three fixed types (nothing, a glider, or a lightweight spaceship) were selected at random, and placed at random positions in a 15x15 square cell array. The GoL evolution rule was run for 1000 steps, which, in most cases (judged by visual inspection) was enough for the system to settle on a stable configuration – which was typically either nothing, a small number of static structures, or a small number of particles in non-colliding tracks. To compute the emergent feature

$V_{t'}$, particles were detected by simply pattern-matching the resulting system state against the known shapes of each particle. We considered five categories: still lifes, oscillators, gliders, lightweight spacesphips, or nothing.

For static structures, we used a single symbol to represent all still lifes, and a single symbol for all oscillators. This particle detector was found to be very effective, with only 2% of runs resulting in unrecognised particles. A total of 5×10^4 independent runs were simulated and, due to the high number of possible states of V_t, to reduce bias we used the quasi-Bayesian estimator by Archer *et al.* [4].

For the boids simulation, $N = 10$ boids were simulated on a torus of side length $L = 200$. Boids are initialised with random positions, speeds, and head angles, and at each timestep each boid $i = 1, ..., N$ is updated according to the equations:

$$
\begin{aligned}
x^i_{t'} &= x^i_t + s^i \cos(\alpha^i_t) \\
y^i_{t'} &= y^i_t + s^i \sin(\alpha^i_t) \\
\alpha^i_{t'} &= \alpha^i_t + a_1\theta_1 + a_2(\pi + \theta_2) + a_3\theta_3 \ ,
\end{aligned}
$$

Where θ_1 is the bearing to the flock's center of mass, θ_2 the bearing to the nearest boid, and θ_3 the mean alignment of all boids within a 20 unit radius. The scalars a_1, a_2, a_3 are the aggregation, avoidance, and alignment parameters, respectively.

The results in Figure 5 in the main text were obtained averaging Ψ across 25 independent runs of 5000 timesteps each, keeping $a_1 = 0.15$, $a_3 = 0.25$ fixed across all simulations. To compute Ψ, we pre-processed the trajectories using the same procedure as Seth [5] (i.e. each boid was described by its distance to the center of the environment and all time series were first-order differenced), and information-theoretic quantities were computed using the non-parametric estimator implemented in the JIDT toolbox [6, 7] using a dynamic correlation exclusion window of 10 samples [8].

To compute the uncertainties and error bars reported in the text and figures we used standard surrogate data methodology: first, system trajectories are time-shuffled to generate one set of surrogate time series, then the quantities of interest (e.g. $\Psi^{(1)}_{t,t'}$) are estimated on the surrogate data, and standard deviations over multiple realisations of the surrogates are reported.

6 ECoG preprocessing and decoding

ECoG signals were preprocessed following the steps presented in the original publication: specifically, the data was notch-filtered to remove line noise and band-pass filtered from 0.1 Hz to 600 Hz, and then downsampled to the same sampling frequency as the MoCap data (120 Hz) [9]. Then, data were divided into a 60/40 train/test split. To build the predictor, the training set was first standardised to zero mean and unit variance, and then it was dimensionality-reduced with a 20-component Partial Least Squares (PLS) regression using the three dimensions of the MoCap as dependent variables. We then trained a non-linear Support Vector Machine (SVM) [10] using a squared exponential kernel predicting the MoCap data from the 20-dimensional PLS scores. SVM and kernel hyperparameters were optimised through 5-fold cross-validation, and a model with the best hyperparameter configuration was trained on the full training set. This cross-validation and optimisation procedure was repeated for each dimension of the MoCap data, resulting in three separate SVMs.[1]

[1]Note that the accuracy of this model is lower than the model presented in Ref. [9], for a precise reason: to predict position at time t, Chao *et al.* use a wavelet transform to obtain features that have represent ECoG from $t-1.1$ s to t. Unlike Chao *et al.*, we work under the constraint of using supervenient features: for the axioms of the theory (in its current form) to hold, V_t needs to be a function *of* $\boldsymbol{X}_t$ *only*. This limits the possibilities for feature extraction, and naturally may result in lower-accuracy decoders.

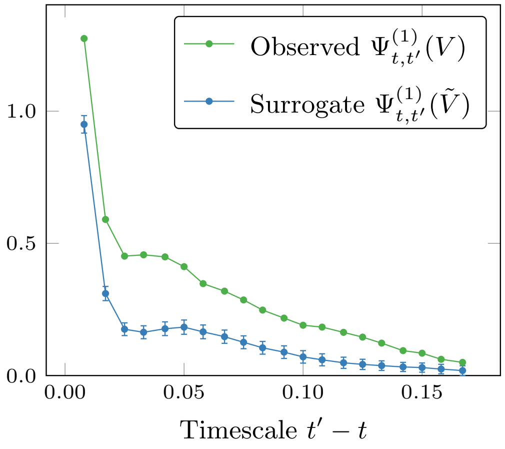

Fig B. Surrogate data test for emergence in monkey ECoG. As a control, we computed the emergence criterion Ψ on a surrogate feature $\tilde{V}$, and found that it yields significantly lower Ψ than the original data (error bars are standard error of the mean over 10 realisations of $\tilde{V}$; see text for details).

The computation of Ψ was performed on the held-out test data set. ECoG signals were standardised and projected onto the PLS latent space (using the means, standard deviations, and projection matrix obtained from the training data), and mutual information terms involved in the computation of Ψ were calculated using the Gaussian estimator implemented in the open-source JIDT toolbox [7].

Additionally, we performed a control using surrogate data to confirm the results were not driven by the autocorrelation or filtering properties of the ECoG, or the regularisation of the SVM. For this, we time-shuffled the wrist position time series, trained an SVM, and evaluated it on a separate (un-shuffled) ECoG test set, resulting in a surrogate feature $\tilde{V}_t$ that preserves the autocorrelation properties of the ECoG but does not meaninfully extract motor information. Then we computed $\Psi^{(1)}_{t,t'}(\tilde{V})$, repeated over 10 realisations (i.e. shuffling and training a separate PLS-SVM 10 times), and found that the observed $\Psi^{(1)}_{t,t'}(V)$ lies significantly above its surrogate, confirming the observed emergence criterion scores over and above what would be expected by the autocorrelation structure of the ECoG (Fig. B).

References

1. Mediano PA, Rosas F, Carhart-Harris RL, Seth AK, Barrett AB. Beyond integrated information: A taxonomy of information dynamics phenomena. arXiv preprint arXiv:190902297. 2019;.

2. Kolchinsky A. A novel approach to multivariate redundancy and synergy. arXiv preprint arXiv:190808642. 2019;.

3. Barrett AB. Exploration of synergistic and redundant information sharing in static and dynamical Gaussian systems. Physical Review E. 2015;91:052802. doi:10.1103/PhysRevE.91.052802.

4. Archer E, Park I, Pillow J. Bayesian and quasi-Bayesian estimators for mutual information from discrete data. Entropy. 2013;15(5):1738–1755.

5. Seth AK. Measuring autonomy and emergence via Granger causality. Artificial Life. 2010;16(2):179–196.

6. Kraskov A, Stögbauer H, Grassberger P. Estimating mutual information. Physical Review E. 2004;69(6):066138. doi:10.1103/PhysRevE.69.066138.

7. Lizier JT. JIDT: An Information-Theoretic Toolkit for Studying the Dynamics of Complex Systems. Frontiers in Robotics and AI. 2014;1:37. doi:10.3389/frobt.2014.00011.

8. Kantz H, Schreiber T. Nonlinear Time Series Analysis. Cambridge University Press; 2004.

9. Chao Z, Nagasaka Y, Fujii N. Long-term asynchronous decoding of arm motion using electrocorticographic signals in monkey. Frontiers in Neuroengineering. 2010;3:3. doi:10.3389/fneng.2010.00003.

10. Bishop CM. Pattern Recognition and Machine Learning. Springer; 2006.
