# Supplementary material for: Reconciling emergences: An information-theoretic approach to identify causal emergence in multivariate data
Source: PLoS Comput Biol. 2020 Dec 21;16(12):e1008289. doi: 10.1371/journal.pcbi.1008289 (PMC7833221; doi:10.1371/journal.pcbi.1008289)
Supplement: Attachment — Submitted filename: response.pdf [file pcbi.1008289.s002.pdf]

Responses to comments from reviewers of paper

Manuscript ID *PCOMPBIOL-D-20-01042*

“Reconciling emergences: An information-theoretic approach to identify causal emergence in multivariate data”

RC *Reviewer’s Comment*

AR Authors’ Response

We would like to thank the editor and the reviewers for their thoughtful work in reviewing our paper. The received comments have helped us substantially improve the revised manuscript. In order to aid the revision process, the modified text in the resubmitted manuscript has been highlighted using colour blue. In addition to the changes mentioned below, we have uploaded the code used to compute all measures of emergence to a public open-source repository, and provided an author summary.

Sincerely,
Fernando, Pedro, Henrik, Anil, Adam, Robin, and Daniel.

Responses to comments from Reviewer 1

RC *What can I say - I re-read this piece a few times and I cannot really find any fault. It deals with what, in my mind, is the most challenging piece of mathematical modeling - the modeling of emergence. The authors achieve this in a very accomplished manner both theoretically and- very importantly, practically. I guess once in a while the only thing left to a reviewer is to congratulate the authors for a masterful submission - and this is the time to do that.*

AR We are thankful to the reviewer for the very encouraging words.

Responses to comments from Reviewer 2

RC *The authors present a new theoretical framework for thinking about the issue of emergence in complex systems. By considering features extracted from a multivariate dynamical system they define two different types of evolving relationship: causal decoupling and downward causation. They provide specific measure based on PID to quantify these properties, and courser level measures to demonstrate their existence with quantities that are easier to compute in practise.*

For a highly technical subject matter the manuscript is very clearly presented and was a pleasure to read. They situate their approach well in comparison to existing techniques from complex systems and the study of consciousness.

AR We thank the reviewer for the positive feedback, and for the very thoughtful comments and suggestions.

RC *Major comments.*

The examples are very nice but I think ECoG data need a null comparison. There is a strong effect of timescale on the measures considered, but neural signals can be strongly autocorrelated with high power in low frequencies and this autocorrelation can change the bias properties of information theoretic measures as a function of frequency / timescale. I think a full treatment of the bias properties of these measures is certainly beyond the scope of this paper, but a simple shuffled control would be enough to demonstrate whether the profiles seen might be influenced by autocorrelation / filtering parameters. For example, the whole analysis procedure could be repeated with the same filtering and cross-validation pipeline, but at the start all the wrist position timecourses used for training should be permuted (e.g. random circular shift or shuffling across trials). This would lead to a classifier V_t that does not extract any meaningful information from the ECoG, but has the same properties induced by autocorrelation, filtering and regularisation. The plots in panel d) could be shown for 1 such random permutation to see if there is any similar dependence on timescale. (or ideally run many times and mean + spread reported, but understand that may be computationally demanding).

AR We thank the reviewer for this interesting suggestion. We had done similar controls before by shuffling both ECoG and wrist position, and the results were conclusive: essentially all correlations vanished, with an average $\Psi^{(1)}$ at short time-scales of around -0.01 and a standard deviations of the order of 10^{-4}. Following the reviewer's suggestion, we did a test shuffling the wrist position but not the ECoG – which, as the reviewer correctly states, accounts for autocorrelations in the ECoG signals. Interestingly, the mean value of Ψ under this null distribution (averaged across 10 repetitions) is non-zero, but is still much lower than the Ψ obtained on the observed data. We have added a paragraph describing the test in Section IV-B, and more details and a figure with the results in Appendix F.

RC *Minor comments.*

It would be nice to have a bit more discussion and motivation of the definition of supervenient feature, as it seems this is a key area where this framework differs from others (discussed nicely P10-11). In discussion P9 supervenient is described as a property "that can be computed from the state of the system". But the definition P3 is stronger than that. Would be nice to have some more motivation where the definition is introduced, perhaps with some intuitive examples of features that would be supervenient, and those that would not meet the definition.

AR Thanks for the excellent suggestion. We have introduced a short subsection II-A (pg. 3) dedicated to supervencience. This subsection presents our definition of supervenience in a more formal manner (following the suggestion of Reviewer 3), comments the motivation behind it, and

briefly discusses some examples.

RC *P3 "information that is provided independently (and hence redundantly) by both of them". Not sure I agree that {1}{2} PID term should be called independent information. In the two-bit copy I think its fair to say each predictor provides information about the target independently, but they are not redundant. Maybe could just say "information that is provided by both of them."*

AR We appreciate the suggestion, as it clarifies the passage. We have changed the text accordingly.

RC *P5 Eqs 5 and 6. Could be clearer for the reader to put the term for $D(k)$ and $G(k)$ directly by the definition, ie "we introduce the downward causation, denoted $D(k)$, and the causal decoupling indices, denoted $G(k)$" and switch the order of equations to match the order they are presented in the text.*

AR We have modified the order of eqs. (5) and (6) to match the text. Also, we thank the reviewer for the suggestion, but we believe that by changing the order of the text one introduces the additional complexity of having an index k that is not been defined until the very end of the sentence. So, while acknowledge that both current and suggested choices have pros and cons, we choose to keep the existent one.

RC *P5. Eq 9. Definition of $Un(k)$ conditioning on multiple terms wasn't clear to me (ie how to relate to definition on p3)*

AR Thanks for pointing this issue. The revised manuscirpt includes the following clarification (footnote 23 in pg. 6, right after Definition 4): "Please note that $\texttt{Un}^{(k)}(V_t; V_{t'}|\boldsymbol{X}_t, \boldsymbol{X}_{t'})$ is information shared between V_t and $V_{t'}$ that no combination of k or less variables from $\boldsymbol{X}_t$ or $\boldsymbol{X}_{t'}$ has in its own."

RC *P8, +/- uncertainty are reported on the numerical results, but not told what these are (s.d., s.e.m., confidence interval etc.) Similarly Fig 5. Error bars not specified.*

AR All error bars are standard deviations computed on surrogate data. We have added clarification remarks for both cases (footnote [37], and in the caption of Fig. 5 in pg. 8), and also a explanation in Appendix E (pg. 17) that we quote here for convenience: "To compute the uncertainties and error bars reported in the text and figures we used standard surrogate data methodology: first, system trajectories are time-shuffled to generate one set of surrogate time series, then the quantities of interest (e.g. $\Psi^{(1)}_{t,t'}$) are estimated on the surrogate data, and standard deviations over multiple realisations of the surrogates are reported."

RC *P9 "linking supervenience to static and causal power to dynamic properties" sentence unclear, add commas or repeat the word properties?*

AR Thanks for pointing this out. We have reworded the sentence (pg. 10) as "Nonetheless, by linking supervenience to static relationships and causal power to dynamical properties, our

framework shows...".

RC *P16: typo "pecifically".*

AR Thanks. The typo has been fixed.

RC *P17 "calculated using ... JIDT". More details of which method and associated options? (JIDT implements a wide range of estimators)*

AR Thanks for pointing this out. Appendix F now clarifies that the method used was a Gaussian estimator.

Responses to comments from Reviewer 3

RC *This study proposed possible information theoretic formulations of causal emergence. Specifically, the study distinguished downward causation and causal decoupling and showed critical conditions for the existence of them both theoretically and practically. What is particularly exciting about this study is the application of PID and Phi-ID in this context, because they clarify and disentangle various existing ideas surrounding the notion of emergence. I'm particularly impressed by the theorems presented here, because they allow us to detect causal emergence when we do not know exact ways to construct features. I do not have major concerns regarding the contents of the paper.*

AR We thank the reviewer for the very positive comments.

RC *I have one general question about causal decoupling. I understand that decoupling exists (i.e. is defined) mathematically in the parity dynamics example. But I wonder whether this can occur in physical interactions which should cover much smaller part of all possible dynamics. So I'm curious to know whether causal decoupling is possible for a dynamics where the dynamics is determined by direct interactions among micro elements (i.e., the state of one element is determined by the states of other elements). Intuitively, if I consider physical implementations of the parity case, I would think we need an additional physical element to directly store the parity of the current state. If this is indeed required, the process itself relies on something like ghost (i.e., not existent, but is used for computation). In other words, what I'm suggesting is that decoupling exists only in mathematics, but not in physics. So I was curious how Gamma behaves in application cases. But the Gamma was not zero, so it may be difficult to find an example of decoupling in real/simulation cases. Could the authors comment on this if they had any thoughts?*

AR The reviewer raises a very interesting point. It is true that the dynamics of the parity case (Example 1 in pg. 2) is not expressible in terms of direct interactions between the micro-elements; which could indeed give the impression that causal decoupling is not realisable by physical systems. Fortunately this is not the case, as shown by our results in the Game of Life (Section IV-A-1, pg. 7-8). Specifically, while the structure of the dynamics of the Game of Life satisfy what one would expect from physical systems (i.e. local interactions between microscopic elements), our results

show that particles exhibit emergent dynamics.

Our revised manuscript includes the following clarificatory remark about this issue (Section II-C-2, pg. 6): "Importantly, the case studies presented in Section IV show that causal decoupling can take place not only in toy models but also in diverse scenarios of practical relevance."

RC *For practical applications, the authors focused on $k = 1$ cases. I understand that this is a pragmatic choice, but I wonder how results may different if we consider $k > 1$ cases. Do we need to consider them to be more precise or is there anything that we may miss and be cautious about?*

AR This is also a really interesting issue, thanks for bringing this up. In general, the value of k determines a different level of microscopic interactions from which a target feature may or not be emergent from. For example, causal decoupling with $k = 1$ means decoupling from individual elements, while decoupling with $k = 2$ means decoupling from all groups of two elements; hence, satisfying causal decoupling for $k = 2$ is more challenging than for $k = 1$. Therefore, in case studies it could be of great interest to explore up to which value of k does the system still exhibit emergence. However, higher values of k require the estimation of information-theoretic properties in higher dimensions, which could require exponentially more data.

As the reviewer said, we decided to focus the applications of this paper in the simpler case $k = 1$, and leave the exploration of $k > 1$ for future publications. Nonetheless, we have added a discussion on these interesting and relevant issues in Section III-A (pg. 6-7), which we quote here for convencience:

"It is worth noticing that the value of k can be tuned to explore emergence with respect to different "scales." For example, $k = 1$ corresponds to emergence with respect to individual microscopic elements, while $k = 2$ refers to emergence with respect to all couples – i.e. individual elements and their pairwise interactions. Accordingly, the criteria in Proposition 1 are, in general, harder to satisfy for larger values of k. In addition, from a practical perspective, considering large values of k requires estimating information-theoretic quantities in high-dimensional distributions, which usually requires exponentially larger amounts of data."

RC *Very minor points: Page 3: A formal definition of a supervenient feature would help clarify what kind of functions are assumed.*

AR Thanks for this suggestion. We have included a new Subsection II-A (pg. 3) dedicated to supervenience, which presents the definition in a more formal manner and also briefly discusses some examples.

RC *Page 5. The order of eq. 5 an eq. 6 should be reversed for consistency with the text.*

AR Thanks for pointing this out. We have reversed the order of these equations to match the corresponding text.

RC *Page 11: "Taken's" should be "Takens'" or "Takens's" as the name is "Takens". Relatedly, Satohiro Tajima had a few papers motivated by Takens/Sugihara embedding to compute integrated information using delay embedding.*

AR Many thanks for these observations. We have fixed the typo, and included a reference to the recent work of Tajima and Kanai.
